# A study protocol to explore the effectiveness and implementation of mobile community outreach services for women who use drugs in Baltimore, Maryland: The SHOUT Study

**DOI:** 10.1371/journal.pone.0336607

**Published:** 2025-12-01

**Authors:** Sewika Sulpe, Catherine Tomko, Emily Clouse, Andrea L. Wirtz, Brian Weir, Nouran El-Ashry, Susan G. Sherman

**Affiliations:** 1 Department of Health, Behavior, and Society, Johns Hopkins University Bloomberg School of Public Health, Baltimore, Maryland, United States of America; 2 Department of Epidemiology, Johns Hopkins University Bloomberg School of Public Health, Baltimore, Maryland, United States of America; PLOS: Public Library of Science, UNITED STATES OF AMERICA

## Abstract

The opioid overdose crisis has continued to affect women who use drugs (WWUD), particularly in urban cities such as Baltimore, Maryland, where fatal overdose rates rank among the highest in the nation. Despite evidence demonstrating the impact of mobile health services in serving underserved populations, few interventions are specifically tailored to meet the unique needs of WWUD. The Sustained Harm Reduction OUTreach (SHOUT) study evaluates the effectiveness and implementation of a harm reduction–based mobile outreach service operated by a community-based organization serving WWUD in Southwest Baltimore. The “intervention group” consists of WWUD recruited within the organization’s catchment area, while the “control group” comprises those recruited from neighborhoods outside of the organization’s catchment area. The study’s three aims are: (1) to conduct in-depth interviews with WWUD (N=12) to assess the feasibility of using a modified respondent-driven sampling (RDS) method; (2) to conduct a prospective cohort study comparing intervention (N=250) and control (N=150) groups over 18 months to assess nonfatal overdose and healthcare access outcomes; and (3) to evaluate intervention implementation using the RE-AIM framework. The study is guided by Andersen’s Behavioral Model for Vulnerable Populations and Rhodes’s Risk Environment Framework. Preliminary findings suggest that a modified RDS approach is both feasible and acceptable among WWUD. Aim 2 will examine the effect of mobile services on reducing nonfatal overdoses by promoting harm reduction practices within participants’ social and physical environments. Aim 3 will incorporate qualitative and cost-effectiveness analyses to contextualize the program’s impact and sustainability. This study addresses critical service gaps for WWUD by integrating and providing low-barrier harm reduction services offered on an accessible mobile van. Findings will inform scalable, community-driven strategies to reduce overdose mortality and improve health equity among structurally vulnerable populations. Strengths, limitations, and plans for results dissemination are discussed.

## Introduction

Following a steady escalation in drug overdose deaths in the United States (U.S.) during the COVID-19 pandemic, national data through 2023 indicate a gradual decline in both fatal and non-fatal overdoses [1]. However, overdose mortality rates remain significantly higher than pre-pandemic levels, underscoring the ongoing severity of the crisis [1–3]. Reports from 2024–2025 indicate that Baltimore, Maryland, has become a critical hotspot in the national overdose crisis, with both fatal and non-fatal overdose rates rising to alarming levels [1,2,4]. The city’s fatal overdose rate is the highest among major U.S. cities, with 170 deaths per 100,000 people between 2018 and 2022—nearly double the rates seen in other large cities and far exceeding the national average of 32.6 deaths per 100,000 in 2022 [4,5]. Non-fatal overdoses have also surged, with opioid-related emergency department visits in Maryland increasing by 13.1% between early 2023 and 2024 [6,7].

Baltimore continues to rank as one of the jurisdictions with the highest fatal overdose rates among African American men [8]. While comprehensive national data on the drivers and disparities of overdoses remain limited, there has been a notable and concerning rise in fatal overdose rates among women who use drugs (WWUD), which have nearly doubled since the early 2000s [3,9]. This persistent rise is spearheaded by several overlapping and reinforcing factors, including the unstable illicit drug market dominated by potent synthetic opioids like fentanyl and nitazenes, deep-rooted socioeconomic disparities (e.g., housing instability, food insecurity, poor sanitation access, and transportation issues), and systemic challenges in addressing addiction [1,2,6,10]. In this complex landscape, WWUD additionally face unique or markedly heightened challenges compared to men that further increase their overdose risk, such as gender-based and intimate partner violence [11–16]. WWUD also encounter barriers to accessing healthcare, engulfed with stigma and discrimination pertaining to substance use [17,18], sex work [19,20], and race or ethnicity [21,22]. These intersecting issues highlight the consistent need for targeted interventions and sustained investment in harm reduction, treatment, and community support to address WWUD’s needs amid the persistent opioid epidemic.

Mobile health services have proven effective in reaching socially disadvantaged and medically underserved communities, facilitating access to care for populations that are traditionally difficult to engage [23,24]. Mobile vans have been successfully used to provide essential HIV prevention services to people who use drugs (PWUD), including sterile syringes, HIV/STI testing, and buprenorphine treatment directly in community settings [25–28]. A recent study found that mobile harm reduction units were more frequently visited by historically marginalized racial and ethnic groups and those experiencing homelessness, suggesting this model may help address systemic barriers to addiction treatment and harm reduction services [29,30]. Despite their effectiveness, mobile health services remain underutilized in the U.S. as a scalable strategy to improve healthcare access, particularly for WWUD, whose needs require trauma-informed and comprehensive harm reduction services [24].

The SHOUT (Sustained Harm Reduction OUTreach) study uses a hybrid effectiveness-implementation design to expand and evaluate mobile outreach services provided by the community-based mobile harm reduction and outreach program serving WWUD in Baltimore City.

### Community partner: SPARC center

The Sex workers Promoting Action, Risk reduction, and Community mobilization (SPARC) Center is a low-barrier drop-by center and mobile outreach program that provides services primarily to WWUD and women who sell sex in Southwest Baltimore. SPARC addresses WWUD’s needs by offering trauma-informed services (e.g., syringe service programs, legal services, fentanyl test strips, naloxone training) that help reduce overdose risk and improve access to essential healthcare services, including reproductive and sexual health care, low-barrier addiction management, and case management services. During Maryland’s shelter-in-place order beginning in March 2020, SPARC pivoted to a “drop-off” model with supplies requested via texts or calls while the brick-and-mortar Center was closed.

### Intervention

Since launching mobile outreach in 2018, SPARC has adapted its services to meet evolving needs, including a 350% increase in encounters during the COVID-19 pandemic. When the Center reopened in Spring 2021, the SPARC mobile program had grown substantially to 1,100 monthly client encounters, representing a 350% increase from prior to COVID-19. Mobile outreach is conducted in South Baltimore and provides a broad range of services, including harm reduction supplies (e.g., naloxone, safer drug kits) and seasonal items (e.g., hand warmers), wound care management, trauma-informed case management, legal assistance, mental health services, and clinical care. Post-pandemic, Mobile SPARC is phasing back into its original mobile service model in addition to delivering pre-ordered bag drop-offs.

SPARC outreach operates independently of SHOUT, the research study aiming to reduce overdose risks and improve healthcare access for WWUD by evaluating Sparc innovative, low-barrier outreach service model. However, SPARC does provide aggregated data to SHOUT for each outreach shift about location and time of their outreach shift; number of encounters; services or supplies provided, and referrals given.

## Materials and methods

**Fig 1:**
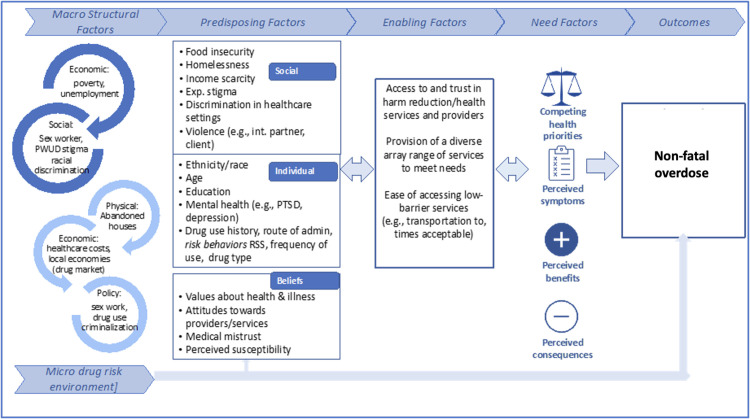
Andersen’s Behavioral Model for Underserved Populations (ABMVP) and Rhodes’ risk environment framework.

The SHOUT study builds on Andersen’s Behavioral Model for Underserved Populations (ABMVP) and Rhodes’ risk environment framework that conceptualizes the drivers of study outcomes. The ABMVP (Fig 1) posits that individual characteristics (e.g., race, drug use) and social factors (e.g., homelessness, food insecurity, violence) serve as predisposing influences that may prompt health-seeking behavior. Enabling factors (e.g., healthcare access, affordability, and nature of services) further shape healthcare-seeking engagements, while need factors, including individuals’ competing health priorities such as perceived overdose risks, are particularly relevant to the underserved population being studied. In alignment with Rhodes’ risk environment framework, the study situates Mobile SPARC’s services within the broader social and physical environment, recognizing how macro- (e.g., drug policy) and micro- (e.g., neighborhood conditions) environmental factors influence health outcomes.

Specifically, the study aims to:

1. Conduct formative research to specifically examine the feasibility of employing a social network-based sampling strategy, modified respondent-driven sampling (RDS), a novel technique for this population.
2. Evaluate the effectiveness of expanding Mobile SPARC on nonfatal overdose over time in WWUD in the intervention (n = 250) compared to those in the control group (n = 150).
3. Conduct an implementation evaluation for the Mobile SPARC intervention using the Re-Aim framework.

The study hypothesizes that factors across the ABMVP framework and Rhodes’ risk environment framework will contribute to the study outcomes through exposure to Mobile SPARC. Mobile SPARC aims to normalize overdose prevention behaviors by providing holistic, nonjudgmental, and client-centered services. By offering these services directly within the participant’s immediate (micro) environment, the intervention will facilitate access to healthcare and create new opportunities for engagement. We further hypothesize that the comprehensive nature of these services will primarily reduce the burden of nonfatal overdoses and secondarily dismantle barriers to accessing broader healthcare services.

This study has received ethical approval from the Johns Hopkins University Bloomberg School of Public Health Institutional Review Board (IRB#00022100, approved 8/31/2022).

### Study timeline

The participant recruitment and data collection for Aim 1 took place between October 12 and October 24, 2023. We completed Aim 1 in February 2024 and began participant recruitment for Aim 2 in March 2024. Recruitment is still ongoing at the control sites, and we anticipate completing baseline recruitment by December 2025. The initial 6-month follow-up at the intervention sites began in January 2025 and will continue concurrently throughout the study period. Once the final 18-month follow-up concludes, we will initiate Aim 3 activities, which will include interviews with SPARC staff, cost analysis, and the integration of the RE-AIM framework into our evaluation process.

### Aim 1

During the study’s formative phase, we conducted in-depth interviews with WWUD to understand their perspectives on peer recruitment methods. Peer recruitment methods are used in recruiting populations that are underrepresented in research; however, the stigma associated with certain identities or behaviors, such as substance use, may make WWUD hesitant to participate; these interviews were intended to inform the contours of Aim 2 sampling in a way that is acceptable to the target population.

#### Recruitment.

Participants were recruited from areas of Baltimore identified as areas of high drug-related activity (see Aim 2 methods for more detail). Study staff drove a mobile van to these locations and informed women appearing to be 18 years or older about the study opportunity. Interested individuals returned to the mobile van for eligibility screening and, if eligible, provided oral informed consent. Recruitment used purposive sampling to capture a broad range of experiences in terms of demographics (e.g., race/ethnicity, age); behaviors (e.g., drug types used, length of drug use); and social context (e.g., experiencing homelessness, strength of social ties). Inclusion criteria for Aims 1 and 2 were: ≥ 18 years; self-identify as a woman; and used opioids (e.g., heroin, pills, fentanyl), crack cocaine, and/or cocaine more than 3 times in the past 3 months.

#### In-depth interviews with WWUD.

Interviews were conducted using a semi-structured guide that explored a range of topics related to peer recruitment methods, including Respondent Driven Sampling (RDS), to examine the appropriateness of using a modified RDS method. Interview questions addressed women’s peer networks, attitudes towards peer referrals within those networks, concerns and strategies to mitigate them, and logistical considerations for recruitment (e.g., incentive type and amount, ways to retain coupons). Participants were compensated with a $50 Visa gift card per interview. Audio recordings of the interviews were transcribed verbatim for data analysis.

This study marked our first use of modified-Respondent-Driven Sampling (RDS) for recruiting within this population, despite our long-standing engagement and prior research with them [11–15,19,31–33]. We originally planned for N = 20 interviews, but reached saturation after N = 12 interviews; therefore, we stopped data collection as no new insights emerged from additional interviews.

#### Data analysis.

Qualitative data management and analysis were conducted using MAXQDA software. All interview recordings were transcribed, translated where necessary, and uploaded to MAXQDA for coding and analysis. Data analysis followed these successive steps: 1) multiple readings of transcripts with memoing to note initial key themes; 2) open coding of selected rich passages; 3) coding of all interviews; 4) grouping, comparison, and categorization of themes by interview; 5) synthesizing and comparing through thematic analysis [34].

## Results

Interview findings suggested that RDS with cash reimbursement would be both appropriate and acceptable for this group. Based on these insights, the team integrated participant feedback into the development of our recruitment protocols. Cash reimbursement emerged as the most practical incentive for the study population. To support participants in remembering and securing their RDS referral information, we employed various tools such as coupons and plastic wristlets with embedded coupon codes. Although we initially had concerns that participants might be reluctant to return for referral compensation, interviews indicated this was not an issue.

### Aim 2

Aim 2 investigates the effect of expanding Mobile SPARC on nonfatal overdoses over time among WWUD in the intervention group (n = 250) compared to those in the comparison group (N = 150). Participants will be recruited and followed over 18 months, with visits scheduled at 6-month intervals. As of October 2025, recruitment is still ongoing.

#### Sample size calculation.

For sample size calculations, power analyses assumed up to 25% loss to follow-up and an average of three follow-up visits per participant over 18 months. For the non-fatal overdose outcome, assuming a cumulative rate of 36% in the control group and lower rates in the intervention group, the study has 85% power to detect RR = 0.56 (or 80% power for RR = 0.58) with N = 400. With N = 300, the detectable RR is 0.50 at 85% power. These estimates suggest that the proposed sample size provides adequate power to detect clinically meaningful effects under realistic follow-up conditions.

#### Geographic zones.

We are recruiting N = 250 WWUD in the two existing geographic zones where Mobile SPARC currently operates in West Baltimore. Mobile SPARC staff mapped their existing outreach route using the Strava app to determine geographic service boundaries and, consequently, the study’s data collection boundaries. N = 150 WWUD are being recruited in ‘control’ areas. Control zones were identified based on high concentrations of 911 calls for overdose and drug arrests from the Baltimore Fire and Police Departments using a publicly available municipal database. These geographic areas in Northwest and East Baltimore are comparable in terms of population and drug activity. However, the control areas lack routine exposure to SPARC and its mobile outreach catchment services, and recent research also indicated that WWUD in these areas rarely report utilizing SPARC’s services [35]. The control areas are served by other health and social services for vulnerable populations. One harm reduction-focused organization serves people who use drugs with mobile outreach services, though our control areas are outside of this organization’s typical catchment area.

In each geographic zone, we first conduct a windshield and walkthrough tour of the recruitment site in pairs of two or three. These tours help validate the appropriateness of zones that were identified using 911 and arrest data. Study staff have forms on which to note how many people in the area appear to be women over 18 years old, pedestrian traffic levels, businesses nearby, and locations to park the RV for recruitment. Based on these observations, the team determines whether the zone is appropriate and identifies an optimal RV parking location.

#### Modified RDS method and network analysis.

Recruitment into intervention and control groups utilizes a modified RDS method. RDS is a non-probability chain referral sampling method that has been widely used to engage populations that lack a sampling frame [36]. The method, including modified versions, has been used in research conducted in Baltimore City [37,38]. Because this method leverages trust and connections within communities, it was refined for use in the National HIV Behavioral Survey (NHBS) among People who inject drugs (PWID) and has continued to be used by the CDC and other investigators to evaluate trends in HIV and other health outcomes among PWID [37,39–47]. We chose this method as PWID are often well-networked and connected to other PWID in close geographic proximity [48], making network-based referrals an optimal recruitment method. Further, RDS often permits reach to individuals who are not engaged in health services.

Modified RDS began with an initial selection of approximately five seeds of well-networked PWUD, selected to maximize variation in ethnicity/race, age, and gender. Candidates were identified in each zone through conversations with staff during formative research (Aim 1). Seeds participate in study activities and refer up to three peers who will, in turn, be invited to participate in study activities and recruit others until the target sample size is reached. RDS is monitored in real-time to ensure that recruitment is progressing, identify and address bottlenecks, and monitor homophily in the study sample. New seeds are initiated if initial seeds are unproductive or recruitment slows. Given the evaluation focus rather than estimation of population prevalence that requires attention to specific assumptions, we used a modified approach that permits eligible individuals who approach the study staff but have not been referred by a seed/recruiter to enter the study as a seed themselves [36,37,49]. Seeds and recruiters receive secondary incentives of $5 per peer referral who completes the baseline survey. Paper and electronic coupons are provided to participants to facilitate referrals, with each coupon containing a unique code that allows for anonymous identification of the seed/recruiter as well as study branding and contact information to call and be screened for study inclusion. A form is established in our REDCap system to track recruitment information and coupon numbers from seeds and recruiters, and is built to mimic the RDS Coupon Manager [50], a common tool used for monitoring RDS.

#### Recruitment.

Once baseline recruitment begins, field staff walk around the area within 1–2 blocks, distributing recruitment cards to women on the street. Interested women are invited to return to the mobile RV to learn more about the study and be screened for eligibility if interested in a more private space.

To enroll in the study, the participants must be over 18, identify as a woman, and have used opioids or cocaine more than three times in the past week. Engagement with Mobile SPARC is not a requirement, as the study aims to assess the program’s community reach and impact. The intervention is introduced sequentially across the two zones, allowing a staggered rollout to manage resources for both SPARC and the study. SPARC services will continue beyond the study period.

#### Data collection.

Baseline and follow-up visits occur on the study van. After screening for eligibility and obtaining written consent, participants: 1) provide detailed locator information including phone numbers, addresses, email, social media accounts, and information for stable contacts; 2) complete a 45–60-minute audio computer-assisted self-interview (ACASI), with an interviewer available for assistance; 3) receive referrals to appropriate services, if desired; and 4) are compensated with a $50 visa gift card for their time. Each participant is assigned a unique ID. Regardless of whether they are in the intervention or control arm, all participants receive snacks and drinks, safer sex supplies (e.g., condoms), and safer drug use supplies (e.g., fentanyl test strips, naloxone).

In addition to standard demographics, the survey captures key outcomes related to overdose experiences and substance use history (past 6 months and 30 days), including type of drug used (or combination of drugs, e.g., fentanyl and cocaine), frequency, and routes of drug administration. Predisposing factors are assessed through socioeconomic status, housing stability, history of arrests, incarceration, and insurance status. Enabling factors include measures of healthcare access (primary care, specialty care, mental health providers, and substance use treatment), mental health status measured using the PHQ-9 for depression [51] and PCL-5 for PTSD [52], medical mistrust using an adapted Medical Mistrust Scale [53], and stigma using the Perceived Stigma of Substance Use [54] and Illicit Drug Use Stigma Scales [55]. Need factors focus on health priorities, comorbid conditions, and perceived care outcomes.

#### Cohort retention.

To maximize cohort retention, we will employ follow–up strategies that have proven effective in similar studies, achieving follow-up rates of 75–80% over 18 months [56]. At each visit, detailed locator information is collected and regularly updated to ensure successful ongoing contact with participants. The locator form captures each participant’s preferred method of communication, such as phone, text, social media, or via a stable contact, as well as their current address, a physical description, and contact information for at least two stable contacts who can receive messages on their behalf. Study staff utilize this information to make follow-up contact using a dedicated study Google Voice number, study email, and phone line.

In addition to remote outreach, staff use public databases, including the Maryland Inmate Locator, Maryland Case Search, and the Maryland Death Certificate Index, to help locate participants who are difficult to reach. Home visits to recorded addresses are also conducted when necessary. Printed study flyers are distributed as appointment reminders, and monthly updates are shared across study social media platforms to inform participants of upcoming follow-up activities in specific areas. To further support retention, staff maintain a consistent presence in recruitment areas throughout the study period.

#### Statistical data analysis.

The primary outcome analysis will examine whether the number of non-fatal overdoses during follow-up is lower for participants at the intervention sites versus the control sites, controlling for the number of non-fatal overdoses prior to baseline and adjusting for baseline covariates through inverse probability of treatment weights (IPTWs). The outcome model will use over-dispersed Poisson regression of counts of non-fatal overdoses adjusted for person-time at risk. Person-time for each participant will be the cumulative time intervals covered by questions about non-fatal overdose across the follow-up assessments. The primary independent variable will be whether a participant was enrolled from an intervention vs. control site, and, as intervention status is not randomly assigned, IPTWs will be used to improve exchangeability [57]. Participants in the intervention zones may differ from participants in the control zone at baseline in important ways that may affect or otherwise be associated with the likelihood of use of harm reduction services during follow-up and non-fatal overdose and other outcomes during follow-up.

IPTWs will be constructed from baseline covariates, including types, routes, and patterns of substance use, substance use treatment history, past overdose, demographics, employment, housing, physical health, mental health, and recent use of different harm reduction services, among other covariates. We will calculate standardized bias for baseline covariates as the difference in means or proportions between participants in the intervention and control sites, divided by the pooled SD for the covariate. Baseline covariates with standardized bias greater than 0.1 will be considered as candidates for bias reduction. Those variables and other baseline variables expected to be associated with the use of harm reduction services and overdose risk will be used as predictors in logistic regression of treatment status to generate IPTWs. We will then evaluate whether the weights have an acceptable distribution (e.g., mean approximately 1 and outliers limited through trimming of weights) and whether they are successful in reducing bias to an acceptable level (<.2 SD). If the IPTWs fail to achieve adequate balance on any baseline covariate for any site, we will consider including that baseline covariate as a fixed effect in the outcome analyses. Finally, we will conduct the outcome analyses with IPTWs to estimate the effect of the treatment vs. control site on cumulative non-fatal overdose and secondary outcomes.

Given that the intervention is hypothesized to affect overdose and other outcomes through the provision of harm reduction services, we will examine whether participants at the intervention sites versus the control sites are more likely to use harm reduction services during follow-up, controlling for use of harm reduction services prior to baseline and adjusting for baseline covariates using mixed effects models with IPTWs [58]. Observations (t) will be nested within participants (i), and the logistic models will include participant-level random intercepts, indicators for each follow-up assessment, group, group-by-follow-up interactions, and other baseline covariates. Whether treatment status is significantly associated with the use of harm reduction services will be evaluated through a simultaneous test of all group-by-follow-up coefficients.

Mixed effects models will also be used to evaluate the association of treatment status with other secondary outcomes, such as use of opioids and stimulants, injection drug use, syringe sharing, use of Test Strips, naloxone possession, intercourse without HIV pre-exposure prophylaxis (PrEP) or condoms, and unmet health or social services needs. The analytic approach will be the same as for the use of harm reduction services, but the link function and distributional assumption will be matched to the specific outcome.

### Aim 3

This aim will assess the implementation of Mobile SPARC through a variety of evaluation activities guided by the RE-AIM framework, examining the domains of Reach, Effectiveness, Adoption, Implementation, and Maintenance. Table 1 presents the definitions of each RE-AIM domain and details the quantitative and qualitative data sources supporting this evaluation.

**Table 1:** RE-AIM framework and data collection.

Framework components		Data sources		
Domain	Definition	Quantitative	Qualitative	
			KII	IDI
**Reach**	Who receives the intervention	Aim 2 results	X	
**Effectiveness**	Effect of intervention on outcomes	Aim 2 results	X	X
**Adoption**	Factors contributing to Intervention uptake	Aim 2 results	X	X
**Implementation**	How/when intervention is implemented	SPARC admin data	X	
**Maintenance**	What is sustained or changed after initial Implementation	Cost analysis	X	

KII, Key Informant Interviews; IDI, In-depth Interview

### Qualitative data sources

#### SPARC staff interviews.

We will conduct semi-structured interviews with Mobile SPARC staff (N = 5–8) to gather insights into each RE-AIM domain. These interviews will focus on staff perceptions of Reach (who uses Mobile SPARC services), Effectiveness (impact of interventions on outcomes), Adoption (reflections on adoption in new neighborhoods and implementation factors influencing adoption), Implementation (process descriptions), and Maintenance (sustainability considerations). Eligible staff will include those with at least one outreach shift in the expanded service areas. Interviews will be conducted immediately post-18-month follow-up and will be audio recorded with consent; otherwise, detailed notes will be taken.

#### WWUD interviews.

We will purposively sample women who use drugs (N = 20) to represent a range of ages, races, and substance use histories. Eligible WWUD participants will have used Mobile SPARC at least once during the study. These in-depth interviews, done at the 18-month follow-up, will explore RE-AIM domains such as Effectiveness (extent to which services met needs), Adoption (benefits and areas for improvement), and Implementation (service interactions). Participants will receive a $40 Visa gift card for participation. Interviews will be audio-recorded, transcribed verbatim, and analyzed for consistency across domains.

#### Qualitative analysis.

Data from interviews will be analyzed thematically based on RE-AIM domains to identify factors influencing Mobile SPARC’s success. Analyses will focus on key aspects of service reach, unmet needs, and implementation facilitators, with findings guiding future Mobile SPARC service enhancements.

### Quantitative data sources

The Aim 2 dataset and SPARC administrative data, as described in the Aim 2 section, will contribute to the RE-AIM analysis to measure the Reach, Adoption, and Implementation aspects.

#### Cost analysis.

We will conduct a cost-effectiveness analysis to evaluate Mobile SPARC’s societal value, following guidelines from the 2nd US Panel on Cost-Effectiveness in Health and Medicine [59]. Using a micro-costing approach, we will assess resources utilized, including staff and participant contributions. Costs will be recorded at two intervention points, with a “best estimate” range to account for variability. We will determine cost-effectiveness by estimating the cost per visit and per person-year, informing effectiveness thresholds.

We will further estimate Mobile SPARC’s impact on health outcomes, utilizing quality-adjusted life years (QALYs) and participant-reported health states. Transmission models will estimate HIV and HCV incidence, informed by recent literature on health costs and QALYs. Outcomes will be evaluated based on societal willingness-to-pay thresholds, with sensitivity analyses examining parameter uncertainties and robustness.

### Data management

An extensive quality assurance and quality control system is developed by the study director and investigators for all study procedures, including recruitment, real-time data checks, and staff observation notes throughout the study period for Aim 2. A database management system maintains and tracks all data, including participant contact information, which is stripped of identifiers, assigning a unique code, and is stored on a password-protected computer drive with limited access to authorized staff.

## Discussion

### Strengths and limitations

The study faces several challenges, one of which is the mobility of the target population, which can make follow-up challenging, even within a six-month period. Building on over a decade of experience working with this community, we will continue to implement innovative and intensive strategies to locate WWUD [56]. This includes extensive use of locator form data and assigning dedicated research staff to focus on participant tracking and retention. Additionally, the study team will closely monitor changes in the local drug market, as well as any policy shifts that could affect participant mobility or impact the logistical aspects of study operations. Further, the presence of other organizations serving people who use drugs in Baltimore may confound findings related to SPARC outreach utilization and overdose outcomes. Our statistical analysis plan attempts to account for exposure to these other harm reduction and drug treatment services, but we cannot eliminate the threat to validity completely.

Despite these limitations, the study is distinguished by several key strengths that enhance its potential impact on WWUD and their access to healthcare. The study design is intentionally structured to reduce logistical barriers through a mobile outreach intervention, supporting the delivery of healthcare services that are available, accessible, and acceptable in the community, meeting participants where they are. This approach is expected to contribute to a reduction in non-fatal overdose rates and improve healthcare access among WWUD in Baltimore City. Further, the study team worked closely with the community partner (SPARC) to design data collection instruments and incorporate their feedback and input into the study design. This strong collaborative relationship benefits the research and WWUD being served by SPARC. Additionally, our rigorous recruitment strategy is designed to strengthen the study’s generalizability, both within Baltimore and across similar urban settings.

### Dissemination plan

Study results will be disseminated through multiple channels to ensure broad impact and accessibility. Academic dissemination will include peer-reviewed manuscripts and presentations at national and international conferences. To ensure results reach study participants and the broader community, we will create plain-language fact sheets highlighting key and relevant findings. These materials will be distributed by SPARC, as well as partner organizations that serve similar populations. Additionally, de-identified study data will be shared via the Inter-university Consortium for Political and Social Research (ICPSR) data repository, as part of our involvement in the Harm Reduction Research Network.

Results from this study will directly inform the scale-up of community-delivered, low-barrier harm reduction strategies, such as naloxone and drug checking strip distribution, that meet WWUD literally and figuratively where they are. Our findings may also have implications for similar populations who face substantial barriers to accessing healthcare, such as people experiencing homelessness, or for whom engaging in traditional overdose prevention efforts is challenging, by providing a model for low-barrier service delivery.
